# A real-time PCR assay for rapid identification of inducible and acquired clarithromycin resistance in *Mycobacterium abscessus*

**DOI:** 10.1186/s12879-020-05686-0

**Published:** 2020-12-10

**Authors:** Meenu Kaushal Sharma, Yanni La, Debra Janella, Hafid Soualhine

**Affiliations:** 1 National Reference Centre for Mycobacteriology, National Microbiology Laboratory, Public Health Agency of Canada, 1015 Arlington St, R3E 3R2 Winnipeg, Canada; 2 Department of Medical Microbiology, University of Manitoba, Winnipeg, Canada; 3 Department of Microbiology, University of Manitoba, Winnipeg, Canada

**Keywords:** *Mycobacterium abscessus*, Real-time PCR, *erm*(41), Clarithromycin

## Abstract

**Background:**

*Mycobacterium abscessus* is a rapidly growing mycobacteria involved in severe infections of the lung, skin, or soft tissue. Macrolides such as clarithromycin are the recommended first line drugs for treatment of *M. abscessus* infections. However, *M. abscessus* has dual mechanisms of resistance to macrolides, making treatment by macrolides difficult. A functional *erm*(41) gene confers for inducible resistance while acquired mutations on the 23S rRNA *rrl* gene confer for constitutive resistance.

**Methods:**

We have developed a real-time PCR assay to detect both inducible and acquired resistance to clarithromycin, and compared the results to traditional *erm*(41) and *rrl* sequencing and phenotypic susceptibility testing using Sensititre™ plates.

**Results:**

Of the total 126 *M. abscessus* isolates tested, truncated *erm*(41) was found in 23/126 (18.3%) of the samples, 27/126 (21.4%) had a T28C mutation in *erm*(41), and 2/126 (1.6%) had an acquired A2058C mutation in *rrl*. The phenotypic results correlated with the expected sequencing results in 121/126 samples (96%). Phenotypic testing compared to real-time PCR resolved 2 of these discrepancies by showing the existence of both *erm*(41) alleles in the isolates that sequencing missed. One culture was found to be mixed with two *M. abscessus* subsp. as per *hsp65* sequencing and 2 isolates had discordance between molecular and phenotypic results. It was presumed that 3 isolates showed discrepancy between sequencing and real-time PCR, but one culture was mixed and other 2 detected both alleles by real-time PCR leading to 100% concordance when compared to sequencing.

**Conclusion:**

In conclusion, real-time PCR is more accurate for detection of both acquired and induced clarithromycin resistance, specifically when mixed genic profiles are present in a sample.

## Background

As per former and accepted taxonomy, the rapidly growing *Mycobacterium abscessus* is subdivided to 3 subspecies *M. abscessus* subsp. *abscessus*, *M. abscessus* subsp. *bolletii*, and *M. abscessus* subsp. *massiliense* which are involved in infections of the skin, lung, as well as infections associated with medical procedures [1, 2]. These infections are commonly treated with macrolides (clarithromycin, azithromycin and erythromycin) but resistance to macrolides makes treatment increasingly difficult [3, 4]. *M. abscessus* infections management may require multi-drug therapy along with intravenous treatment for several months. Intravenous agents such as amikacin are commonly associated with side effects in patients [5]. Identifying resistance earlier can determine the best suited treatment. According to the Clinical Laboratory Standard Institute (CLSI), drug susceptibility testing for clarithromycin require an incubation time of up to 14 days to assess inducible resistance [6].

Natural and acquired resistance to clarithromycin are due to the erythromycin ribosomal methyl transferase, *erm*(41), and the gene encoding a 23S peptidyl transferase in the large 23S ribosomal subunit, *rrl*, respectively [7–9]. Inducible resistance occurs naturally with a functional *erm*(41) gene [7]. However, a common trait of *M. abscessus* subsp. *massiliense* isolates is that they have a 274-bp deletion in *erm*(41), making it non-functional, hence susceptible to macrolides [7]. Thus, *M. abscessus* subspecies identification and determination of macrolide susceptibility are useful in the planning of the appropriate treatment. Another mutation, the T to C mutation at position 28 on *erm*(41) also leads to a non-functional erythromycin ribosomal methyl transferase [7]. When inducible resistance occurs, drug susceptibility testing will show susceptibility in vitro at day 3 but will later develop resistance up to 14 days post-incubation. Clinically acquired resistance to a macrolide occurs from a spontaneous mutation at positions 2058 and 2059 on the *rrl* gene of 23S, which causes an alteration to the drug binding pocket of the protein [8, 9]. Constitutive resistance will result in a high minimum inhibitory concentration (MIC) at day 3 of susceptibility testing. As reported by Vester et al. (2001), acquired mutations at positions 2057 and 2611 on *rrl* can also result in low level resistance due to being in close proximity to the action centre [10]. These mutations are outside the focal point of macrolide interaction but can still disrupt the structure of the drug binding pocket, reducing its ability to inhibit the ribosome [10].

Sequencing of 16S and *hsp65* (heat shock protein 65) genes was also used to identify the different subspecies of *M. abscessus*. Sequencing of *erm*(41) and *rrl* was done and probes for real-time PCR were designed to look for possible mutations that would cause resistance to clarithromycin. A real-time PCR assay was designed to run under a single condition which would greatly decrease the turnaround time of 14 days using phenotypic testing, to just a few hours to predict resistance to clarithromycin. Real-time PCR assay was also compared to the sequencing method currently used in our laboratory.

## Methods

### Identification of *M. abscessus* subspecies

*M. abscessus* isolates identification was performed using 16S gene sequencing and the subsp. identification was done using sequencing of the *hsp65* gene. PCR amplification was done in a 50 μL reaction volume consisting of 47.5 μL premix (1 μM forward primer, 1 μM reverse primer), 0.25 μL of Qiagen *Taq* DNA polymerase (Qiagen, Hilden, Germany), and 2.5 μL of template DNA (Table 1). Amplification was done using the Applied Biosystems Veriti® 96-Well Thermal Cycler (Thermo Fisher Scientific, Applied Biosystems, Grand Island, NY). Thermocycler conditions for *hsp65* were 95 °C for 5 min, 45 cycles of 95 °C for 1 min, 60 °C for 1 min, and 72 °C for 1 min, followed by 72 °C for 5 min. The PCR products were purified using PCRClean DX magnetic beads (Aline Biosciences, Woburn, MA). Sequencing was done using Applied Biosystems 3730*xl* DNA Analyzer (Thermo Fisher Scientific, Applied Biosystems, Grand Island, NY). Sequences were assembled using Lasergene SeqMan Pro (DNAStar, Inc., Madison, WI) and sequence comparison was done using BioNumerics 7.6.2 software. Reference strains *Mycobacterium abscessus* subsp. *abscessus* ATCC 19977^T^, *Mycobacterium abscessus* subsp. *bolletii* CCUG 50184^T^, and *Mycobacterium abscessus* subsp. *massiliense* CCUG 48898^T^ were used.

**Table 1:** Primers used for sequencing of *hsp65*, *erm*(41) and *rrl* of the *M. abscessus*

Primer	Target Gene	Sequence	Annealing Temp (°C)	Reference
TB11	*hsp65*	5′-ACCAACGATGGTGTGTCCAT-3’	60	[11]
TB12	*hsp65*	5′-CTTGTCGAAGGCCATACCCT-3’	60	[11]
ermF	*erm(*41)	5′-GACCGGGGCCTTCTTCGTGAT-3’	60	[12]
ermR1	*erm(*41)	5′-GACTTCCCCGCACCGATTCC-3’	60	[12]
erm41–2	*erm(*41)	5′-GGATTCCGGCGTCAAGAGACTC-3’	60	[9]
erm41–3	*erm(*41)	5′-CGAGCCCGCCCTACCAAGTCAC-3’	60	[7]
erm41–4	*erm(*41)	5′-CCGGCCCGTAGCGTCCAATG-3’	60	[13]
erm41–5	*erm(*41)	5′-ACTCCCCTGAGCGA ACAC-3’	60	[7]
19F	*rrl*	5′-GTAGCGAAATTCCTTGTCGG-3’	55	[14]
21R	*rrl*	5′-TTCCCGCTTAGATGCTTTCAG-3’	55	[14]
SP1	*rrl*	5′-CCTGCACGAATGGCGTAACG-3’	55	[15]
SP2mod	*rrl*	5′-CACCAGAGGTTCGTCCGTC-3’	55	[15]

### Antimicrobial susceptibility testing

Phenotypic susceptibility testing for clarithromycin was done using Sensititre™ RAPMYCO AST plates (Trek Diagnostics, Thermo Fisher Scientific, Oakwood Village, OH) according to the manufacturer instructions. Plates were examined for the drug MICs on day 3 (and day 5 if growth in positive control well was not adequate) of incubation then further incubation at 30 °C was done if day 3 or 5 results showed sensitivity. Plates were examined for inducible resistance on day 7, 10 and 14 of incubation.

### Sequencing of *erm*(41) and *rrl* genes

PCR amplification was done in a total reaction volume of 50 μL which consisted of 25 μL Amplitaq Gold® 360 Mastermix (Thermo Fisher Scientific, Applied Biosystems, Foster City, CA), 22.5 μL forward and reverse primers (final concentration of 1 μM each), and 2.5 μL of template DNA for both *erm*(41) and *rrl* genes. Primers ermF and ermR1 were used to amplify the *erm*(41) gene while primers 19F and 21R amplified the *rrl* gene (Table 1). Thermocycler conditions for amplifying *erm*(41) were 95 °C for 7.5 min for the initial denaturation, followed by 35 cycles of 95 °C for 30 s, 60 °C for 30 s, and 72 °C for 30 s followed by a final extension of 72 °C for 10 min. Conditions were the same for *rrl* except with an annealing temperature of 55 °C instead of 60 °C. The PCR products were purified using PCRClean DX magnetic beads. Sequencing primers are shown in Table 1. Sequencing and analysis were done as described above for *hsp65* gene.

### Real-time PCR assay

A 96-well plate assay was designed based on a modified protocol from Shamira Shallom and colleagues (2015) [11]. The probe, Absc-chel 16S, was used for identification, *erm*(41)_probe1 was used to detect full-length *erm*(41), SNPs on position 28 of *erm*(41) were detected using probes *erm*(41)T28 and *erm*(41)C28, SNPs on position 2058 of *rrl* were detected using probes 23S_A2058 and 23S_C2058 (Table 2). Probes for *rrl*, 23S_A2058 and 23S_C2058, initially consisted of LNA (locked nucleic acids) from the Shallom protocol, however, the *rrl* probes used in this experiment were modified. This change would see a lower melting temperature for these probes and therefore, the 5′ end of these probes were extended by 7 base pairs using Mabs5 23S rRNA (GenBank accession number EU980535.1) to ensure the same melting temperature of around 60 °C. Probes were made to a working concentration of 2.5 μM in a mix with their corresponding primers (Table 2) which were diluted to 10 μM. Reactions were done in a total reaction volume of 25 μL consisting of 12.5 μL TaqMan® Fast Advanced Master Mix (Thermo Fisher Scientific, Life Technologies, Austin, TX.), probes final concentration of 250 nM and forward and reverse primers at final concentration of 1 μM each, and 3.75 μL of template DNA. Each sample was run in duplicates with positive control for all wild type and mutants i.e., 16S, full length *erm*(41), T28T in *erm*(41), T28C in *erm*(41), A2058A in *rrl*, and A2058C in *rrl* and negative control (no sample DNA). Real-time PCR was done using the Applied Biosystems QuantStudio 3 Real-Time PCR System (Thermo Fisher Scientific, Applied Biosystems, Grand Island, NY). Thermocycler conditions included an initial denaturation of 95 °C for 3 min, 40 cycles of 95 °C for 30 s, 60 °C for 30 s, and 72 °C for 30 s.

**Table 2:** Probes and primers used in the real-time assay for *M. abscessus*

Target		Sequence	Reference
**16S**	Absc-chel 16S	5′-6FAM-ACC ACA CAC TTC A-MGB-NFQ-3’	[16]
	F Primer16S	5′-ATAAGCCTGGGAAACTGGGTCTA-3’	
	R Primer16S	5′-CCACACCGCAAAAGCTTT-3’	
**Full length** ***erm*(41)**	*erm*(41)_probe1	5′-6FAM-TGC TAG CCG TCG AGC TGC ATC C-QSY-3’	[17]
	F PrimerTR	5′-TCAGGGGAGTTCGTTGTGGAT-3’	
	R PrimerTR	5′-TCTTCCTCGGCAAACCGT-3’	
***erm*(41)**	*erm*(41)T28	5′-HEX-CCA + G + T + G GGG C-IABkFQ-3’	[12]
	*erm*(41)C28	5′-6FAM-CCA + G + C + G GGGC-IABkFQ-3’	
	F PrimerE28	5′-GAGCATGGGCATATTCATGATGG-3’	
	R PrimerE28	5′-TGAGCGAACACCGGATTCG-3’	
***rrl***	23S_A2058	5′-6FAM-CGGCAGGACGA*A*AAGACCC-BHQ1–3’	[12]
	23S_C2058	5′-6FAM-CGGCAGGACGACAAGACCC-BHQ1–3’	
	F PrimerR2058	5′-GCGAAATTGCACTACGAGTAAAG-3’	
	R PrimerR2058	5′-CCTATCCTACACAAACCGAACC-3’	

*MGB* minor groove binder, *HEX* hexachlorofluorescein, *6FAM* 6-carboxyfluorescein, *IABkFQ* Iowa Black fluorescent quencher, *BHQ1* Black Hole quencher, *NFQ* Non-fluorescent Quencher, +, LNA bases

## Results

The *hsp65* sequences were compared using Bionumerics version 7.6.2 (Applied Math, Belgium). Sequential differences in *hsp65* were used to distinguish the three members of *M. abscessus* as described in Kazue Nakanaga et al. (2014) [18]. It was found that in our 126 samples, 94 were identified as *M. abscessus* subsp. *abscessus*, 7 *M. abscessus* subsp. *bolletii*, 23 *M. abscessus* subsp. *massiliense*, and 2 showed the coexistence of two mixed DNA populations belonging to *M. abscessus* subspecies.

Clarithromycin susceptibility testing using microbroth dilution assay found that of the 126 isolates, 3 were resistant on day 3 (2 *M. abscessus* subsp. *abscessus*, 1 *M. abscessus* subsp. *massiliense*), 76 were inducibly resistant (70 *M. abscessus* subsp. *abscessus*, 5 *M. abscessus* subsp. *bolletii*, 1 *M. abscessus* subsp. *massiliense*). One isolate with intermediate result (*M. abscessus* subsp. *abscessus*) was repeated with an updated phenotypic result showing susceptible result giving a total of 47 susceptible results (22 *M. abscessus* subsp. *abscessus*, 2 *M. abscessus* subsp. *bolletii*, 21 *M. abscessus* subsp. *massiliense,* 2 mixed by *hsp65* sequencing) (Table 3). Inducible resistance is characterized by an MIC of 8 or greater observed on day 7 and up to day 14 after an initial result of susceptibility at day 3 [19].

**Table 3:** Clarithromycin Minimum Inhibitory Concentration (MIC) interpretations for *M. abscessus* isolates

	MIC Interpretation			
***hsp65*** Identification	S	IR	R	Total
*M. abscessus* subsp*. abscessus*	22^a^	70	2	94^a^
*M. abscessus* subsp*. bolletii*	2	5	–	7
*M. abscessus* subsp*. massiliense*	21	1	1	23
*M. abscessus* subspecies mixed	2	–	–	2
Total	**47**	**76**	**3**	**126**

S, Sensitive (MIC ≤2); IR, Inducible Resistance (MIC ≥8 on day 7 or later of susceptibility testing); R, Resistant (MIC ≥8 on day 3 of susceptibility testing); ^a^2 mixed cultures of *M. abscessus* subsp. *abscessus,* each found to have both wild type T28 and mutation C28 in *erm*(41)

### Sequencing of *erm*(41) and *rrl* for mutations

Sequencing found that 23 samples had the 274-bp deletion on *erm*(41). Twenty-two of which were found to be in the subspecies *M. abscessus* subsp. *massiliense*. One was in a mixed population containing *Mycobacterium abscessus* subsp. *massiliense* (Table 4)*.* Twenty-seven strains had the T28C mutation in *erm*(41) (24 *M. abscessus* subsp*. abscessus*, 2 *M. abscessus* subsp*. bolletii*, and 1 mixed) and 2 strains harboured a A2058C mutations on *rrl* (1 *M. abscessus* subsp*. abscessus* and 1 *M. abscessus* subsp*. massiliense*) (Table 4). Five strains (*M. abscessus* subsp. *abscessus*) had discrepant results which had phenotypic results that did not match with the mutation found with sequencing, which have been explained in the discussion section.

**Table 4:** Comparisons of mutations found in *erm*(41) and *rrl* for each subspecies of the *M. abscessus* using sequencing and real-time PCR

Phenotypic Result			Sequencing Results			Real-time PCR Results		
Subspecies	# of Samples		Full-length/truncated ***erm***41	***erm***41 position 28	***rrl*** position 2058	Full-length/truncated ***erm***41	***erm***41 position 28	***rrl*** position 2058
***M. abscessus*** **subsp*. abscessus***								
	67	IR	Full	T	A	Full	T	A
	1	R^a^	Full	T	A	Full	T	A
	1	S^a^	Full	T	A	Full	T	A
	1	R	Full	T	C	Full	T	C
	21	S	Full	C	A	Full	C	A
	2	IR	Full	C	A	Full	T + C	A
	1	IR^a^	Full	C	A	Full	C	A
***M. abscessus*** **subsp.** ***bolletii***								
	5	IR	Full	T	A	Full	T	A
	2	S	Full	C	A	Full	C	A
***M. abscessus*** **subsp*. massiliense***								
	21	S	Truncated	T	A	Truncated	T	A
	1	R	Truncated	T	C	Truncated	T	C
	1	IR	Full	T	A	Full	T	A
^b^ ***M. abscessus***								
	1	S	Full	C	A	Full	C	A
	1	S	Truncated	T	A	Full	C	A

^a^ Discrepant phenotypic result to what was expected with real-time results

^b^ *M. abscessus* subspecies with two *hsp65* gene sequences

### Real-time PCR assay

**Fig. 1:**
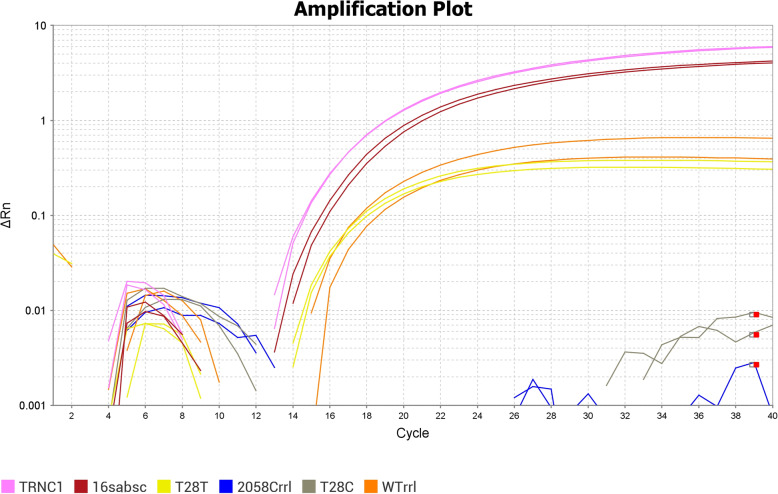
Example of the amplification plot for a sample showing detection of a full-length *erm*(41) with the T allele and a wildtype *rrl* gene (Probe TRNC1 detects full-length *erm*(41) and allows us to determine if the gene is truncated or full-length, 16sabsc verifies *M. abscessus* identification and near neighbors, T28T and T28C detects the 2 alleles on position 28 of *erm*(41), and 2058Crrl and WTrrl for alleles on *rrl*)

Our real-time assay was optimized by modifying Shallom 2015 protocol. Our method ran all of the targeted mutations of a sample on a single plate and under the same PCR conditions which include detection of full-length *erm*(41). Shallom et al. used a SYBR green quantitative method separate from their probe based one for detecting full-length *erm*(41). Additionally, we used a probe for species confirmation in our assay, making our assay far more informative in a single run. The probes for the *rrl* targets originally consisted of LNA bases, but due to availability, ours consist of regular DNA bases. The probes’ length was then changed from 12 to 19 base pairs to ensure a matching melting temperature with other reactions on the plate. This has impact on the real-time PCR conditions as we intend to multiplex this assay at a later date. Black Hole quencher probe tags were also used in place of Iowa Black fluorescent tags for the modified *rrl* probes. These modified probes were locally produced in our facility and therefore more cost effective and allowed the assessment of different probe and conditions. Results of the real-time assay showed amplification for all our targets using the aforementioned probes under the same cycling conditions. Successful detection in our *rrl* targets informs us that LNA bases in probes is not necessary to perform this assay. Crossing threshold (CT) values were found to range between 16 and 24 cycles for different reactions (Fig. 1). Samples were run in duplicates and CT values of duplicates showed minimal variability of one CT with orange color having slightly more variability between duplicates.

When real-time PCR assay was compared to sequencing, one discrepant sample had a truncated *erm*(41) gene with the T allele with sequencing, but real-time PCR showed detection of a full-length gene and a C allele. Both methods found no mutations on position 2058 of the *rrl* gene. This strain was found to be mixed with *M. abscessus* subsp. *abscessus* and *M. abscessus* subsp. *massiliense* as per *hsp65* sequencing data. Two isolates of *M. abscessus* subsp*. abscessus* had discordant sequencing and phenotypic results, with both T and C alleles of *erm*(41) amplified with real-time PCR and only T28C mutation with sequencing. Hence, only sequencing showed discrepancy to the phenotypic results but real-time PCR did not as the latter detected both alleles. Real-time PCR results resolved all these three discrepancies.

## Discussion

Clarithromycin is an important antibiotic against members of the *M. abscessus* [3]. Resistance to macrolide can be predominantly correlated with two genes, *erm*(41) and *rrl* in *M. abscessus*. The full *erm*(41) gene that is without the 274 bp deletion and T at position 28 will result in a functional erythromycin ribosomal methyl transferase and hence show inducible resistance [7]. Extended periods of incubation in the antibiotic will induce resistance in *M. abscessus* isolates that have an inducible resistance genic profile. An acquired point mutation on positions 2058 and/or 2059 of *rrl* will result in high level resistance against clarithromycin by altering the drug binding domain of the 23S rRNA [8, 9]. These are characteristically shown by resistance (MIC ≥8) on day 3 of antimicrobial susceptibility testing and hence indicate constitutive resistance.

Phenotypic susceptibility testing was done by broth microdilution and samples were incubated for up to 14 days [6]. Initial testing found that 79 (62.7%) samples were resistant of which 76 (60.3%) showed inducible resistance. Forty-six were initially shown to be susceptible at day 3; however, one sample with an initial intermediate result was repeated and showed to be sensitive for a total of 47 (37.3%) sensitive samples. For this intermediate sample, molecular methods found it to have a T28C mutation so a susceptible result was expected and overgrowth in the initial phenotypic testing may be the cause for the intermediate reading. In the 2 strains that were determined to be mixed by *hsp65* sequencing, both were susceptible to clarithromycin.

Sequencing of *erm*(41) and *rrl* identified 27 strains (21.4%) with a T28C mutation, 23 (18.3%) had a truncated *erm*(41) gene and 2 (1.6%) with the A2058C mutation on *rrl*. The 274-bp deletion on *erm*(41) was identified in only *M. abscessus* subsp. *massiliense* and 1 mixed *M. abscessus* sample containing *M. abscessus* subsp. *massiliense*. This deletion is characteristic of *M. abscessus* subsp. *massiliense* as reported by Hee-youn Kim et al. (2010) which is why clarithromycin treatment is found to be more effective against infections with *M. abscessus* subsp*. massiliense* than the other subspecies that contain the functional full-length gene [20]. One sample in our study that was identified as *M. abscessus* subsp*. massiliense* was found to possess a full-length *erm*(41) gene by both sequencing and real-time PCR. Shamira Shallom and colleagues (2013) have also identified *M. abscessus* subsp*. massiliense* isolates with full-length *erm*(41) in which they suggest is probably due to horizontal transfer [21].

Sequencing results of *erm*(41) and *rrl* were 96.0% (121/126) concordant with the phenotypic results, with 5 isolates of *M. abscessus* found to have phenotypic results discordant with the molecular results. Two isolates of *M. abscessus* subsp. *abscessus* showed inducible resistance phenotypically, but had the T28C mutation found with sequencing for an expected result to clarithromycin susceptibility. For these two isolates, both T and C alleles on position 28 of *erm*(41) were detected in the real-time PCR assay, showing that both alleles were present and that the real-time PCR method uses a probe based detection and hence was more sensitive than sequencing. Our sequencing method only detected the predominant C allele for this sample as in general, a sequencing method will more frequently utilize DNA template containing a predominant allele. However, real-time PCR can detect both most and less predominant alleles in low DNA concentrations. Another isolate showed an inducible resistant result phenotypically without a corresponding allele. This could not be resolved when both phenotypic and molecular assays were repeated and is likely due to another mutation outside the gene fragments used in this study. Efflux pump ability has been shown to be directly involved with clarithromycin resistance [22]. One isolate that showed a phenotypic susceptible result lacked correlation with genotypic mutations and also could not be resolved when repeated testing showed reproducibility on all assays. This is perhaps due to unknown mechanism that renders the isolate susceptible to clarithromycin. Extending the search to look for additional mutations did not show *erm*(41) C19T and hence evidence of susceptibility is lacking [23]. The fifth discordant result was resistant on day 3 of susceptibility testing indicating an acquired resistance to clarithromycin, but had no mutations found on positions 2058 of *rrl* with sequencing. This is suspected to be caused by a point mutation elsewhere on *rrl* that would affect the drug binding pocket. The discrepant strains that could be resolved by *hsp65* or real-time PCR had their clarithromycin phenotypic susceptibility testing repeated with 3 of the final results staying reproducible to the initial results. There were no additional mutations in the *rrl* gene such as A2056G, A2057G, A2080C, G2068A, A2269G, G2281A, as the sequence matched wild type [10, 23].

A real-time assay was developed to assess resistance in *M. abscessus* isolates by targeting mutations that were found in sequencing. The assay was built upon the protocol in Shallom et al. (2015) for *erm*(41) and *rrl* with primer and tag modifications that allowed us to perform amplification under a single cycling condition; and included additional probes for 16S identification and full length *erm*(41) detection [12, 16, 17]. A probe for detecting the 16S gene of *M. abscessus* was used to confirm that samples were members of the *M. abscessus* and verified that *erm*(41) is present [16]. Discrimination of full-length and truncated *erm*(41) was done by having a probe and the reverse primer hybridize the region of the gene that is deleted. Amplification indicates a full-length *erm*(41) gene [17]. Two sets of probes were used to detect the mutant and wild type alleles for each position 28 of *erm*(41) and position 2058 of *rrl* [12]. With all of the included probes in our assay, identification and mutation detection can be determined for a sample after a single real-time PCR run. Compared to the 3–14 days needed for susceptibility testing, this modified real-time PCR method has significant impact in a clinical laboratory as it is much faster and helps in determining the best treatment. Samples were run in duplicates and were found to have similar CT and ΔRn values (Fig. 1) for each target, showing the high reproducibility of the assay and had CT values ranging from 16 to 24 for all reactions. Real-time results were compared to the sequencing data to examine adequate amplification in our assay. The real-time results confirmed the sequencing results in our samples. One sample showed a truncated *erm*(41) gene in sequencing, but detection of the full-length gene was observed with real-time PCR. Upon repeating *hsp65* sequencing for this discrepancy, it was found to be mixed with both *M. abscessus* subsp. *abscessus* and *M. abscessus* subsp. *massiliense*. Hence, both test results were correct. Two samples detected both alleles of *erm*(41) by real-time PCR whereas sequencing only detected single C allele. The phenotypic results for these samples showed inducible resistance, indicating that the T allele was present and only real-time PCR was sensitive enough to detect it compared to sequencing. Hence, real-time PCR was superior to sequencing as it requires smaller DNA concentration and may detect both mutation profiles in a single test. Real-time PCR also has an advantage over the sequencing method done in our laboratory as it provides rapid test turnaround times, is less labour intensive with satisfactory test accuracy. The resulting output is a simple negative or positive for a real-time reaction i.e., looking for amplification vs interpreting sequencing chromatograms and hence less data screening. To summarize, even though 3 results were presumed discrepant between phenotypic and real-time PCR (97.6%; 123/126); 1 was found to be mixed by *hsp65* sequencing and 2 samples detected both T28 and C28 *erm*(41) alleles by real-time PCR. This meant that in the presumed discrepant isolates, 100% concordance between sequencing and real-time was seen due to the real-time results. Hence, real-time PCR demonstrated slight advantage over traditional sequencing assay.

## Conclusion

In conclusion, a real-time detection assay shortened turnaround times compared to phenotypic susceptibility testing and traditional sequencing. Probes for detecting A2058G and A2059G mutations on *rrl* were not appropriately evaluated in our study since incidence of *rrl* mutations were low. However, *rrl* was implemented in our assay for future testing to detect possible mutations that may be responsible for constitutive resistance. Multiplexing this assay could also result in conservation of reagents and hence decreased cost while running more samples on one plate/run. Whether by use of sequencing or by real-time PCR assay, there is advantage in detecting *erm*(41) variants to predict sensitivity of *M. abscessus* isolates to clarithromycin, hence impacting treatment outcomes.

## Data Availability

The nucleotide datasets generated and/or analyzed during the current study are available in the NCBI under BankIt submission number 2402752 and accession numbers MW275120 - MW275245.

## Abbreviations

PCR: Polymerase chain reaction
rRNA: ribosomal ribonucleic acid
DNA: deoxyribonucleic acid
LNA: locked nucleic acids
CT: Threshold cycle
ΔRn: Normalized reporter value minus baseline
MIC: minimum inhibitory concentration
CLSI: Clinical Laboratory Standard Institute
